# Longitudinal Association Between Media Use and Development in Preschool: Moderation by Maternal Education

**DOI:** 10.1111/apa.70519

**Published:** 2026-03-26

**Authors:** Judith Eggeling, Christof Meigen, Juliane Ludwig, Wieland Kiess, Tanja Poulain

**Affiliations:** ^1^ LIFE Leipzig Research Center for Civilization Diseases, Leipzig University Leipzig Germany; ^2^ Department of Women and Child Health, Hospital for Children and Adolescents and Center for Pediatric Research (CPL) Leipzig University Leipzig Germany; ^3^ German Center for Child and Adolescent Health (DZKJ), partner Site Leipzig/Dresden Leipzig Germany

**Keywords:** child development, cognitive and language outcomes, electronic media use, maternal education, TV exposure

## Abstract

**Aim:**

This study examined associations between children's use of electronic media and maternal education at age 3, and developmental outcomes and changes (cognitive, language, motor, socio‐emotional) one year later.

**Methods:**

Included were 109 participants of the LIFE Child cohort (Germany). Development was assessed at two time points (mean age = 3.3 years at t1, 4.3 at t2) using the standardised ET 6–6‐R test. Electronic media use (modern media and TV) and maternal education were measured at t1 through parental questionnaires. Linear regression analyses were conducted, adjusting for age, sex and developmental scores at t1.

**Results:**

High TV use (> 1 h/day) at t1 and lower maternal education were significantly associated with poorer cognitive skills at t2. Additionally, high TV use predicted poorer language skills, but this effect appeared only in children whose mothers had low or medium education; no such association was observed when maternal education was high. The use of modern media at t1 showed no significant association with developmental outcomes at t2.

**Conclusion:**

Early TV exposure may negatively affect cognitive and language development. Children from families with lower educational backgrounds appear particularly vulnerable, highlighting the need for targeted media education and guidance.

## Summary

– Little is known about how parental education moderates associations between early media use and later cognitive and linguistic development.
– In this study, high TV use at age 3 was linked to poorer cognitive and language skills one year later, especially in children whose mothers had low or medium education.
– Less educated families should be supported in teaching their children about media use to prevent negative effects on child development.

**Abbreviations**
ET 6–6‐R: Entwicklungstest 6 Monate bis 6 Jahre—Revision (Development Test 6 Months to 6 Years—Revision)
h/day: Hours per day
SES: Socioeconomic Status
t1: Time point 1 (baseline assessment)
t2: Time point 2 (follow‐up assessment)
TV: Television

## 1 Introduction

Children's development is influenced by multiple environmental factors, e.g., socioeconomic status (SES) [1], with maternal education emerging as the strongest predictor [2]. Lower maternal education has been shown to be associated with both poorer cognition [3] and poorer language development [4]. These effects are suggested to be mediated by structural (e.g., leisure affordability), psychosocial (e.g., coping strategies, family support) and behavioural factors (e.g., physical activity, media use) [5].

In families with a lower SES, children's exposure to electronic media is especially high [6]. Several studies have linked early media exposure to developmental problems. For instance, exposure to electronic media was negatively associated with vocabulary size in infants and toddlers [7, 8]. In contrast, other studies suggest that high‐quality media content [9] might have positive effects on linguistic and socio‐emotional development. Regarding cognitive development, overall screen time has been associated with negative outcomes [10]. While certain types of content (e.g., educational screen time) may be beneficial, other types of content (e.g., shows/movies/videos) [11] may have negative effects on cognitive development.

Despite increasing research on media exposure, longitudinal studies examining the role of both SES, particularly maternal education, and media use in child development remain scarce. Maternal education is a key determinant of developmental outcomes, yet the extent to which it may moderate the associations between media exposure in early childhood and developmental outcomes is not well established. This study analysed associations between maternal education and electronic media use at age 3 with cognitive, language, motor and socio‐emotional outcomes one year later. The longitudinal study design enables the examination of developmental trajectories and temporal relationships regarding the assessed characteristics. We differentiated between TV use and modern media and assessed development with a standardised test. Based on previous findings, we expected that high media use and low or medium maternal education would relate to poorer cognitive and language skills, with stronger effects in lower‐education groups.

## 2 Patients and Methods

### 2.1 Participants

The data were collected within the LIFE Child study [12], a longitudinal cohort study conducted at Leipzig University. It investigates healthy child development from birth to young adulthood and aims to improve understanding of non‐communicable diseases. Healthy children without chromosomal or syndromic diseases are recruited until age 16 through local health institutions. Most participants come from Leipzig, a major city in eastern Germany. All participants are invited to attend follow‐up visits, taking part once per year. The study protocol was designed in accordance with the Declaration of Helsinki and was approved by the Ethics Committee of the Medical Faculty of Leipzig University (477/19‐ek). All parents provided informed written consent before the participation of their child.

**FIGURE 1:**
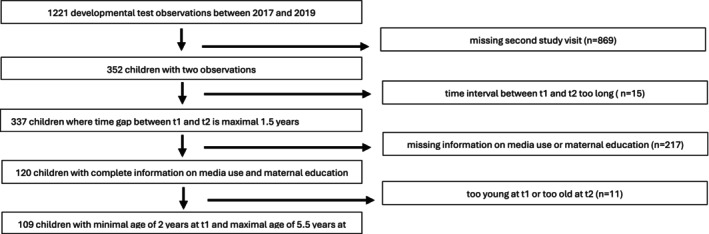
Flow diagram illustrating the inclusion and exclusion of children.

For the present project, data were collected between 2017 and 2019 (to exclude the COVID‐19 pandemic). In this time period, a total of 1221 development tests were performed in 2‐ to 5‐year‐old children. For further analyses, the sample was restricted to children with evaluations at two different time points (t1 and t2) with a time interval of 1.0 to 1.5 years in between (*n* = 337) and with available information on media use and maternal education at t1. After applying these criteria, the final sample included 109 children (50 boys, 59 girls) aged on average 3.4 years (range 2.0–4.4) at t1 and 4.4 years (range 3.0–5.4) at t2 (See Figure 1).

This longitudinal sample comprising healthy, typically developing children aged 2–5 provides a well‐suited population for investigating associations between maternal education, media use and early developmental outcomes.

### 2.2 Measures

#### 2.2.1 Development Test

Child development was tested at t1 and t2 using the test “ET 6–6‐R” (“Development test 6 Months to 6 Years ‐ Revision”) [13]. The test can be applied in children aged 6 months to 6 years and assesses the general level of development in the areas of cognition, language, motor skills and socio‐emotional development. The reliability and validity of the test were shown previously [14, 15]. For each area of development, the number of correctly performed test items was transformed to age‐specific percentile ranks (mean = 50). Percentile ranks indicate the percentage of children in the age‐matched reference population who achieved the same or a lower score than the particular child. A percentile rank of 16 corresponds to an IQ of 85.

#### 2.2.2 Media Use

Children's media use was measured using a parental questionnaire. Parents were asked to report on their child's use of TV/video, laptop/tablet/computer (with and without internet) and mobile phone (with and without internet) on a normal weekday and a normal weekend day. Parents were instructed to exclude the time when the child is not using the screen, e.g., when just listening to music.

Response options ranged from 0 to 4 (“0”: not at all, “1”: approx. 30 min/day, “2”: 1–2 h/day, “3”: 3–4 h/day, “4”: more than 4 h/day). For further analysis, we categorised TV use and the use of modern media (laptop/tablet/computer and mobile phone) into high or low/normal use. For TV use, usage times of 1 h per day or more (response options 2–4) were categorised as high, following current recommendations for a maximal screen time of 1 h per day for 2‐ to 5‐year‐old children [16]. Use of modern media was considered high if at least one device was used for 30 min per day or longer (response options 1–4). These categorisations facilitate meaningful comparisons while considering established developmental guidelines and the generally low exposure to modern media in the sample. Use on weekdays or weekends was assessed separately.

#### 2.2.3 Maternal Education

Maternal education was assessed by asking a parent to indicate the highest school and professional education of the participant's mother. Information on both was combined to a score ranging from 1 to 7, where higher scores indicate higher education [17]. For further analysis, we contrasted high education (University degree, score > 6) and low/medium education (scores 1–6). This cut‐off for higher education was chosen as the University degree represents a standardised indicator of higher educational attainment in Germany. Due to the overall high maternal education, mothers with low education were underrepresented. To enable meaningful statistical analyses, we decided to combine low and medium education groups.

### 2.3 Data Analysis

All analyses were conducted using R, version R 4.3.3 [18]. Data were described in terms of mean values and standard deviations (for continuous variables) or frequencies and percentages (for categorical variables). Associations between variables were assessed using linear regression analyses. The percentile ranks in the different development areas (cognition, language, motor skills, socio‐emotional development) at t2 or the difference in percentile ranks between t1 and t2 were included as dependent variables. In a first step, TV use (during the week and on the weekends separately), use of modern media (during the week and on weekends separately) and maternal education, all assessed at t1, were included separately as independent variables. In a second step, we investigated interactions between TV use or the use of modern media (on the one hand) and maternal education (on the other hand). All associations were adjusted for child sex and age at t1. Associations with percentile ranks at t2 were additionally adjusted for percentile ranks at t1. All associations were considered statistically significant if the *p*‐value was < 0.05. Interactions were only reported if they were statistically significant.

## 3 Results

### 3.1 Description of the Study Sample

Socio‐demographic characteristics, development scores in the different areas and media use characteristics are summarised in Table 1.

**TABLE 1:** Characteristics of the study sample (*n* = 109).

		T1	T2
Sociodemographic characteristics			
Age	Mean (sd)	3.4 (0.7)	4.4 (0.7)
Sex	N (%) male	50 (46%)	
Maternal education	N (%) highest	63 (58%)	
Development (percentile ranks)			
Cognition	Mean (sd, range)	44.6 (27.7, 0.1–99.0)	46.1 (27.2, 0.1–99.7)
Language	Mean (sd, range)	59.5 (25.9, 1.0–84.1)	64.3 (24.7, 2.3–84.1)
Gross motor	Mean (sd, range)	35.1 (23.9, 0.4–90.0)	34.5 (25.6, 1.0–97.7)
Fine motor	Mean (sd, range)	45.0 (27.0, 1.0–95.2)	45.1 (25.7, 0.4–95.2)
Socio‐emotional	Mean (sd, range)	56.6 (29.9, 0.1–97.7)	64.0 (25.8, 4.8–97.7)
Media use			
TV week	N (%) high	15 (13.8%)	—
TV weekend	N (%) high	39 (35.8%)	—
Modern media week	N (%) high	16 (14.7%)	—
Modern media weekend	N (%) high	36 (33.0%)	—

### 3.2 Associations Between Child Media Use and Maternal Education at t1 and Development at t2

As shown in Table 2, long TV use (> 1 h/day) on the weekend at t1 was significantly associated with poorer cognitive skills and poorer language skills at t2. Long TV use during the week was significantly associated with poorer language skills only. A high maternal education at t1 was associated with better cognitive skills but not with other aspects of child development.

**TABLE 2:** Associations between media use and maternal education at t1 and development at t2.

Characteristics at t1		Percentile ranks at t2				
		Cognition	Fine motor	Gross motor	Language	Socio‐emotional
TV weekend	b	**−12.6**	−6.5	−3.4	**−9.3** ^**a**^	−1.3
	95% CI	**−22.7, −2.5**	−17.0, 4.0	−14.1, 7.3	**−18.9, 0.2**	−11.7, 9.0
	p	**0.012**	0.207	0.523	**0.048**	0.791
TV week	b	−5.4	−6.8	−12.6	**−13.9** ^**a**^	−6.3
	95% CI	−18.9, 8.0	−20.8, 7.2	−26.7, 1.5	**−26.3, −1.5**	−19.7, 7.1
	p	0.413	0.323	0.071	**0.025**	0.339
Modern media weekend	b	−3.8	−0.1	−3.8	2.8	−0.6
	95% CI	−13.6, 6.1	−10.4, 10.2	−14.3, 6.7	−6.4, 12.2	−9.2, 10.5
	p	0.437	0.979	0.464	0.530	0.896
Modern media week	b	0.6	−2.1	−5.1	6.0	−10.6
	95% CI	−12.7, 13.9	−15.8, 11.6	−19.0, 8.8	−6.0, 18.1	−23.5, 2.6
	p	0.936	0.758	0.453	0.308	0.099
Maternal education	b	**11.1**	4.3	−5.7	7.5	7.6
	95% CI	**1.8, 20.4**	−5.5, 14.1	−15.6, 4.2	−1.4, 16.3	−1.9, 17.1
	p	**0.017**	0.374	0.240	0.088	0.104

*Note:* All associations are adjusted for chid sex, age, and development scores at t1. Bold values indicate significant associations (*p* < 0.05).

^a^ Significantly moderated by maternal education: negative association only if maternal education was low/medium, not when mat ernal education was high.

**FIGURE 2:**
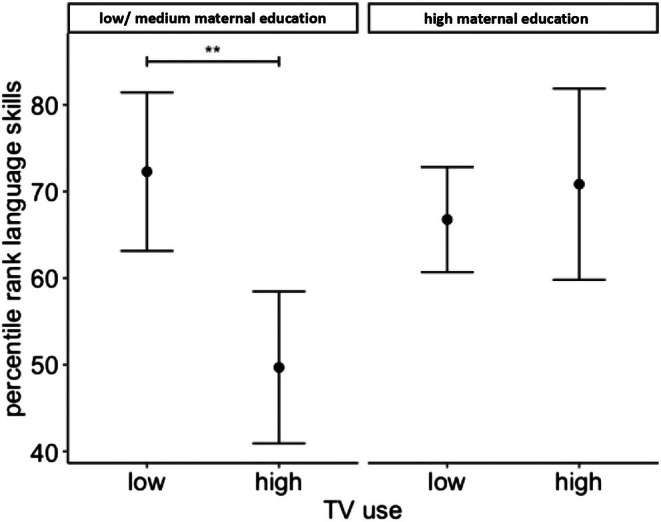
Effect plot illustrating the association (+95% Cl) between TV use on weekends at t1 and language development at t2 in families with low/medium (left) and high (right) maternal education (*n* = 109). High TV use was defined as > 1 h/day. **p < 0.01.

Regarding language skills at t2, the analyses revealed interactions between long TV use and maternal education at t1. This interaction was significant for TV use on the weekend (*p* = 0.006, see Figure 2) and marginally significant for TV use during the week (*p* = 0.067). The interactions showed that high TV use was associated with poorer language skills one year later if maternal education was low/medium (b_week_ = −20.8 percentile ranks, 95% Cl −34.3, −7.3, *p* = 0.002, b_weekend_ = −23.4 percentile ranks, 95% CI −39.0, −7.8, *p* = 0.004) but not if maternal education was high (b_week_ = 4.4 percentile ranks, 95% Cl −8.5, 17.4, *p* = 0.487, b_weekend_ = −1.2 percentile ranks, 95% CI −19.1, 16.6, *p* = 0.892).

In contrast to the use of TV, the use of modern media at t1 was not significantly associated with the development results at t2, and the associations were not moderated by maternal education (all *p* > 0.05).

### 3.3 Associations Between Child Media Use and Maternal Education at t1 and Changes in Development From t1 to t2

**FIGURE 3:**
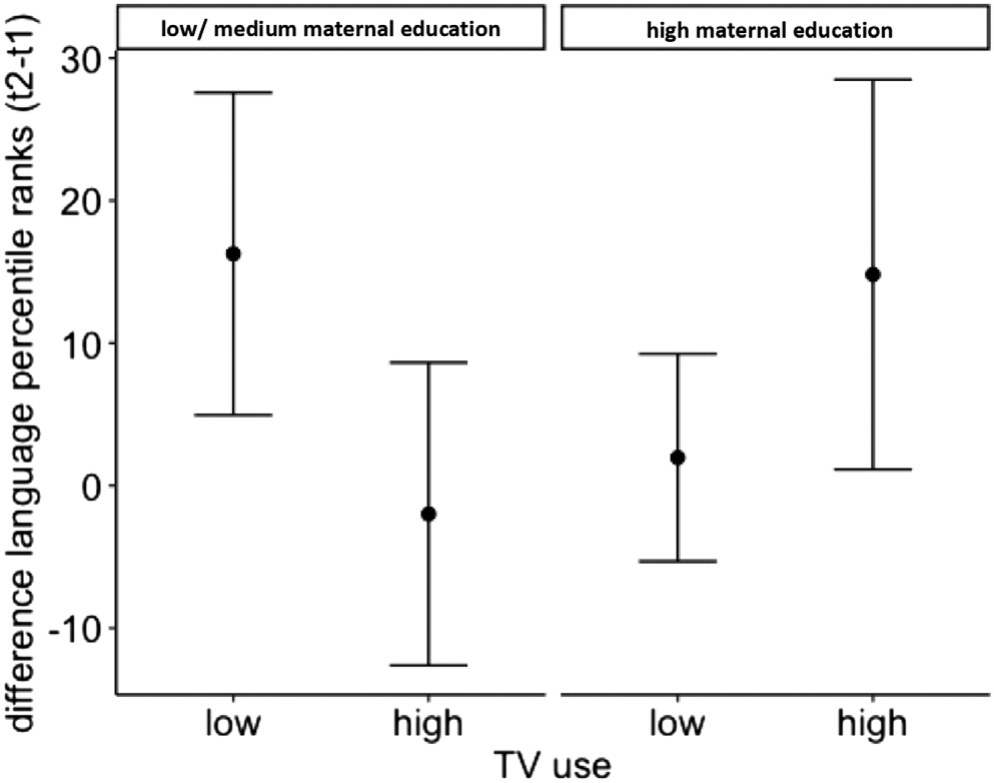
Effect plot illustrating the associations (+95% Cl) between TV use at the weekend at t1 and changes in language development between t1 and t2 in families with low/medium (left) and high (right) maternal education (*n* = 109). High TV use was defined as > 1 h/day.

When investigated separately, neither TV use nor maternal education showed significant associations with the changes in developmental parameters from t1 to t2 (see Table 3). However, regarding changes in language development, the analyses revealed a significant interaction between TV use on the weekend and maternal education at t1 (*p* = 0.012). When maternal education was low/medium, high TV use at t1 was associated with a marginally significant poorer change/increase in language scores from t1 to t2 (b = −15.1 percentile ranks, 95% Cl −31.4, 1.2, *p* = 0.061) (see Figure 3). As shown in the left part of Figure 3, children of mothers with low/medium education who showed a long TV usage time at t1 showed nearly no change in language skills from t1 to t2, while children who showed low/normal TV usage times at t1 showed an increase. When maternal education was high, the opposite trend was observed (see right part of Figure 3), but this association was also not statistically significant (b = 12.8 percentile ranks, 95% Cl −2.8, 28.3, *p* = 0.096). The analyses revealed no significant interaction between TV use during the week and maternal education (*p* > 0.05).

**TABLE 3:** Associations between media use and maternal education at t1 and differences in development between t1 and t2.

Characteristics at t1		Change in percentile ranks (t2‐t1)				
		Cognition	Fine motor	Gross motor	Language	Socio‐emotional
TV weekend	b	−3.7	−3.4	−1.8	−3.4	6.8
	95% CI	−15.5, 8.0	−16.2, 9.5	−14.5, 11.0	−16.2, 9.5	−5.5, 19.2
	*p*	0.521	0.595	0.780	0.595	0.261
TV week	b	−0.5	−5.5	−8.7	−3.4	−1.0
	95% CI	−16.3, 15.2	−22.6, 11.7	−25.6, 8.2	−16.2, 9.5	−17.6, 15.5
	*p*	0.944	0.517	0.298	0.595	0.899
Modern media weekend	b	−1.3	−0.5	−1.5	9.0	−0.1
	95% CI	−12.8, 10.3	−13.1, 12.2	−14.0, 11.0	−1.5, 19.6	−12.3, 12.1
	*p*	0.822	0.938	0.803	0.084	0.990
Modern media week	b	7.5	2.2	−1.7	8.1	−12.3
	95% CI	−7.7, 22.8	−14.6, 18.9	−18.3, 14.9	−6.0, 22.3	−28.3, 3.6
	*p*	0.317	0.793	0.838	0.244	0.118
Maternal education	b	4.1	−1.1	−7.7	−0.4	−0.9
	95% CI	−6.8, 15.0	−13.1, 10.8	−19.5, 4.0	10.6, 9.7	−12.5, 10.6
	*p*	0.445	0.847	0.183	0.933	0.869

*Note:* All associations are adjusted for child sex and age.

For modern media, we observed no significant associations with developmental change between t1 and t2 (see Table 3) and no significant interaction with maternal education.

## 4 Discussion

This study investigated the association between the use of electronic media and maternal education at 3 years of age and child development one year later. Maternal education in our sample was rather high. Regarding the use of electronic media, children aged 3 or 4 years are recommended to use screens for a maximum of one hour per day [16]. In our cohort, more than 10% exceeded this time on a normal weekday, and more than 30% exceeded it on a weekend day, only by watching TV. The use of modern media, in contrast, was very infrequent. This finding suggests that children aged 3 or 4 years old are mainly passive media users, as already suggested in previous studies [19]. The use of interactive media might become popular only later [20]. Regarding the associations of early media use and maternal education with child development one year later, the analyses revealed a significant association of maternal education with cognitive, but not with language, motor or socio‐emotional skills. This is surprising, as previous studies reported several associations [21, 22, 23]. A possible explanation for this could be the small sample size and the few families with low maternal education. Regarding the use of electronic media, the results of our analyses suggest that, overall, early TV use is related to cognitive and language development one year later. This finding is in line with previous studies [24, 25]. One possible reason for this association might be a reduction of social interaction due to higher media exposure. A lack of social interaction has been shown to negatively affect cognition and language [26]. For motor skills, other behaviours, e.g., physical activity [27], might have a more important impact than media use. However, it is surprising that socio‐emotional development was not associated with media use in our study, as other studies showed this relation [28, 29]. In this context, it is important to acknowledge that socio‐emotional skills were assessed via parental reports. Therefore, the results on this scale might be more biased than the results on the other scales.

The lack of an association between the use of modern media and child development might mainly be explained by the overall low usage of these media in our sample.

The main finding of this study is that the association between TV use and language development was moderated by maternal education. In children of mothers with low or medium education, high TV use on weekends (and on weekdays) was linked to poorer language skills one year later. While the language skills of children with low/normal use of TV improved within one year, this improvement could not be observed in children who initially showed high TV use. In children from mothers with a high education, in contrast, TV use was not significantly associated with language skills or changes within one year. In less educated families, the negative effect of early media use may be less able to be compensated for, e.g., through physical activities, reading, language stimulation, and other educational opportunities, than in educated families. In addition, families with higher educational backgrounds may select educational content more frequently and accompany television viewing, while children from socially disadvantaged families may watch less educational TV programs, even without supervision. Importantly, the association between high TV use and cognitive development one year later was not moderated by maternal education, suggesting that the negative effect of high TV use is similar in all social strata.

Stronger associations between weekend TV use and child development may reflect the greater free time available. On weekdays, work and kindergarten leave little time for activities of all kinds, while weekends reveal whether families are more likely to pursue active/educational or rather passive/less educational activities, such as media use.

### 4.1 Strengths and Limitations

The longitudinal design, the assessment of different types of media use and the investigation of maternal education as a moderator represent strengths of the study. However, some weaknesses have to be acknowledged. Media use of children was evaluated by their parents. Parental reports are subjective and might be biased (e.g., social desirability). Furthermore, the sample size was rather small. Finally, children from less educated households were underrepresented in our sample. This limits the generalisability of the study findings to the general population.

## 5 Conclusion

This finding suggests that families with low or medium educational backgrounds need special support/education, e.g., on the subject of media education.

In consideration of these findings, targeted educational campaigns could help inform families, especially those with lower educational backgrounds, about the potential negative effects of excessive TV/media exposure on early cognitive and language development. In addition, support programs might be introduced to reduce the negative effects of high media use and improve the cognitive and language skills of children. Interventions like this could help to reduce educational disparities and promote more equal development opportunities.

## Author Contributions

**Judith Eggeling:** methodology, formal analysis, visualization, writing – original draft. **Christof Meigen:** visualization, writing reviewing. **Juliane Ludwig:** investigation, writing reviewing. **Wieland Kiess:** conceptualization, supervision, funding, acquisition, project administration, resources, writing reviewing. **Tanja Poulain:** conceptualization, supervision, methodology, visualization, writing reviewing.

## Funding

This work was supported by the LIFE Leipzig Research Center for Civilization Diseases at the University of Leipzig. The LIFE Center is funded by the European Union through the European Social Fund (ESF) and the European Regional Development Fund (ERDF), as well as by the Free State of Saxony under the Excellence Initiative.

## Conflicts of Interest

The authors declare no conflicts of interest.

## Data Availability

Research data are not shared.
